# Strengthening mental health care in Somalia through artificial intelligence: opportunities, challenges, and a roadmap for implementation

**DOI:** 10.3389/fdgth.2026.1965858

**Published:** 2026-09-29

**Authors:** Nur Adam Mohamed

**Affiliations:** Department of Psychiatry, Mogadishu Somali-Turkiye Recep Tayyip Erdogan Training and Research Hospital, Mogadishu, Somalia

**Keywords:** artificial intelligence, digital mental health, global mental health, health system strengthening, mental health systems, Somalia

## Abstract

Mental health needs in Somalia are substantial, yet access to timely, high-quality care remains constrained by workforce shortages, limited infrastructure and financing, fragmented services, weak health information systems, and persistent sociocultural barriers. Artificial intelligence (AI) may complement mental health system strengthening in this fragile and resource-constrained setting. This Perspective examines potential applications of AI across Somalia's mental health system and considers the associated implementation, ethical, governance, and equity challenges. Potential applications include AI-assisted screening, clinical decision support, telepsychiatry, digital interventions, predictive analytics, surveillance, health-system planning, and research. Responsible adoption will require locally representative data, appropriate infrastructure and governance, workforce capacity, sociocultural adaptation, equitable access, and sustainable financing. This Perspective proposes a phased roadmap progressing from infrastructure and governance to pilot testing, capacity building, implementation research, and conditional national scale-up. AI should augment rather than replace mental health professionals and remain integrated within broader health-system investment. With local adaptation, community engagement, and continuous evaluation, AI could contribute to more accessible, equitable, resilient, and human-centered mental health care in Somalia.

## Introduction

Mental disorders are leading causes of disability worldwide, contributing to premature mortality, reduced quality of life, and substantial social and economic consequences (1–3). Affecting hundreds of millions across all regions and age groups, conditions including depression, anxiety disorders, schizophrenia, bipolar disorder, and substance use disorders contribute substantially to the global disease burden (1, 4). They also adversely affect education, employment, productivity, and social functioning, burdening individuals, communities, and health systems (5, 6). Mental health is recognized as integral to universal health coverage and sustainable development, yet access to timely, affordable, and high-quality care remains inadequate, particularly in low- and middle-income countries (LMICs) (7).

The mental health treatment gap—the disparity between service needs and access to adequate care—is particularly pronounced in fragile and conflict-affected settings, where high needs coincide with disrupted health systems and limited capacity (8–10). Shortages of trained mental health professionals, insufficient investment, weak health infrastructure, persistent stigma, and limited integration of mental health into primary care collectively constrain the availability and delivery of appropriate mental health services (11–13). As a result, many individuals remain undiagnosed or untreated (13), often seeking care only after substantial deterioration or relying on informal or traditional support (10, 12). Reducing this gap remains a global public health priority and central to equitable mental health care (8).

These pressures are particularly evident in Somalia. Decades of conflict, political instability, climatic shocks, displacement, and economic hardship have increased mental health needs while severely constraining service capacity (11, 12). Despite growing awareness, specialized psychiatric care remains limited by workforce shortages, inadequate infrastructure, fragmented services, and sociocultural stigma (11, 12). A young and rapidly growing population, increasing urbanization, and recurrent humanitarian crises may further increase mental health needs and place additional pressure on already constrained services (11, 14, 15), underscoring the need for innovative, scalable, and contextually appropriate strategies to strengthen Somalia's mental health system.

Recent advances in artificial intelligence (AI) have generated considerable interest in health-care delivery, including mental health services (16). AI encompasses computational techniques such as machine learning, natural language processing, computer vision, and large language models that can analyze complex health data, automate routine tasks, support clinical decision-making, and facilitate remote care (17). Potential psychiatric applications include symptom screening, clinical decision support, suicide risk prediction, telepsychiatry, digital psychoeducation, and population-level surveillance (16, 18, 19). Although many remain under evaluation, these technologies may extend the capacity of the mental health workforce, particularly where specialist services are limited (16, 20).

For Somalia, AI may support task-sharing, extend services to underserved areas, and strengthen health information systems and planning (18, 20–23). However, evidence generated elsewhere may not be directly transferable to the Somali context, and implementation requires careful attention to local infrastructure, data availability, algorithmic bias, regulatory oversight, privacy and security, cultural acceptability, and equity (20, 24, 25). Without appropriate safeguards and local validation, AI could reinforce existing disparities.

This Perspective examines the potential role of AI in strengthening Somalia's mental health system and narrowing its treatment gap. It examines potential applications, implementation constraints, ethical considerations, and policy priorities, and presents a phased roadmap for responsible integration. The analysis aims to inform national decision-making and wider discussion of human-centered mental health-system strengthening in fragile and resource-constrained settings.

## Literature identification and selection

Relevant literature was identified through targeted, non-systematic searches of PubMed and Google Scholar, supplemented by institutional publications and reports from the World Health Organization, the Federal Ministry of Health of Somalia, and the African Union. Searches were conducted throughout the development and revision of the manuscript rather than within a predefined search period. Sources were selected according to their relevance to artificial intelligence, digital mental health, and psychiatric service delivery. Somalia-specific evidence was prioritized where available, while evidence from other resource-constrained settings and the broader international literature was used to address gaps in the local evidence base.

## The mental health system in Somalia: current landscape and persistent challenges

Decades of armed conflict, political instability, climatic shocks, displacement, and socioeconomic adversity have shaped Somalia's mental health landscape, contributing to increased population vulnerability to mental health conditions while constraining the health system's capacity to respond (11, 12). Rapid urbanization, demographic growth, and changing social structures have introduced additional psychosocial stressors, particularly among adolescents and young adults (11, 14). Demand for mental health services has consequently increased despite severely limited resources (11).

Mental disorders represent an important public health challenge in Somalia. Available evidence indicates a substantial burden of depression, anxiety disorders, PTSD, psychotic disorders, and substance use disorders (13, 26–28), reflecting the cumulative effects of conflict, humanitarian crises, poverty, and displacement. Although nationally representative epidemiological data are scarce, studies across diverse population groups consistently demonstrate high psychological distress (28–30), indicating substantial unmet mental health needs.

Despite growing recognition of mental health as a public health priority, Somalia's mental health system faces substantial structural and service-delivery constraints (11). Specialist mental health services remain extremely limited, largely reflecting severe workforce shortages, with inadequate capacity even in major urban centers and greater access barriers in rural and remote communities (12, 13, 31). At the primary-care level, service delivery is affected by shortages of trained personnel, limited integration of standardized mental health screening and assessment, inconsistent availability of essential psychotropic medications, and weak referral pathways (11, 12, 31). The introduction of WHO mhGAP and greater governmental and non-governmental engagement have supported progress, but implementation remains uneven and service coverage insufficient (11, 12).

Workforce shortages and unequal distribution remain major barriers. Somalia has few psychiatrists, psychologists, psychiatric nurses, social workers, and other mental health professionals relative to its population (11, 12, 31), with specialists predominantly concentrated in major cities (12). These shortages constrain early diagnosis, timely intervention, long-term follow-up, and multidisciplinary care while increasing pressure on existing specialists. Primary care physicians and general practitioners often provide mental health services despite limited specialized psychiatric training (32), highlighting the need for scalable approaches that support non-specialist providers without compromising care quality.

Service delivery is also restricted by inadequate infrastructure and financing. Dedicated psychiatric facilities remain scarce, community-based services remain underdeveloped, and electronic health information systems supporting continuity of care and population-level surveillance are largely absent (11, 12, 31). The historically low priority assigned to mental health in national financing has restricted investment in workforce development, service expansion, research, and digital infrastructure (11), limiting the provision of integrated, continuous, and person-centered care.

Access to formal services is also shaped by sociocultural factors. Stigma, cultural beliefs, misconceptions, and discrimination may delay engagement with formal care, while traditional and religious healing practices often provide important support and serve as initial points of contact (11, 12). Delays in accessing formal mental health care may contribute to prolonged illness, greater disability, and poorer outcomes. Collaboration among formal health services, community leaders, and religious stakeholders may improve access to appropriate care.

Policy and service planning are further hindered by a weak evidence base. Research is predominantly cross-sectional, geographically restricted, and focused on specific populations (27–30), while longitudinal research, implementation studies, service evaluations, and nationally coordinated mental health information systems remain scarce. The scarcity of electronic records and routine surveillance restricts monitoring, quality improvement, and evidence-informed decision-making (11, 31). Strengthening research capacity and health information systems is therefore essential for policy development, resource allocation, and intervention evaluation.

Collectively, these challenges demonstrate the complexity of strengthening mental health care in fragile and resource-constrained settings. Workforce expansion and greater financial investment remain essential, but conventional approaches alone are unlikely to substantially reduce Somalia's treatment gap in the foreseeable future. Innovative strategies are therefore needed to extend services, support non-specialist providers, facilitate early identification, strengthen health information systems, and improve population-level surveillance. In this context, AI could complement broader mental health system strengthening (20) by augmenting existing services, improving care efficiency and quality, and expanding equitable access when implemented within appropriate ethical, regulatory, and clinical frameworks (20).

## Artificial intelligence applications for strengthening mental health care in Somalia

For Somalia, the most relevant AI applications are those that could address specific gaps in access, workforce support, information systems, and service planning (17, 19). Its appropriate role would be to support clinicians through decision support, task automation, data analysis, and tailored care (33, 34). Given Somalia's severe workforce shortage, carefully selected applications may extend service reach, support existing providers, strengthen surveillance, and inform planning and resource allocation (18, 35, 36). These applications could complement efforts to strengthen Somalia's mental health system, where specialist services remain severely limited and unevenly distributed. Potential applications of artificial intelligence for strengthening mental health care in Somalia are summarized in Table 1.

**Table 1:** Potential applications of artificial intelligence for strengthening mental health care in Somalia.

Mental health system challenge	Potential AI application	Specific AI contribution	Potential impact
Severe shortage of mental health professionals	AI-assisted clinical decision support systems	Apply machine learning or natural language processing to analyze clinical information and symptom descriptions and provide clinician-reviewed support for assessment, diagnosis, risk evaluation, management, and referral alongside WHO mhGAP guidance.	Improved diagnostic support, clinician confidence, and access to mental health care
Limited access to specialist services in rural and remote areas	AI-enhanced telepsychiatry	Support remote consultations through natural language processing for clinician-reviewed summaries and language assistance, and machine learning to detect possible deterioration from longitudinal symptom data. Videoconferencing and routine scheduling remain conventional digital-health functions.	Greater access to specialist care and continuity of treatment, particularly in underserved areas
Delayed identification of mental disorders	AI-assisted digital screening	Use machine learning or natural language processing to analyze multiple clinical inputs or free-text responses and support risk stratification and referral prioritization.	Earlier identification and referral, with potentially reduced treatment delays
Limited mental health literacy and persistent stigma	Conversational AI and digital psychoeducation platforms	Use natural language processing to interpret Somali-language input and generate adaptive, context-sensitive psychoeducation and self-management guidance.	Improved mental health literacy, greater openness toward mental health care, and increased help-seeking
Limited support for community health workers	AI-assisted decision support for task-sharing	Analyze patient information and symptom descriptions to provide context-sensitive, clinician-reviewed guidance and follow-up recommendations for non-specialist providers.	Greater consistency in community-based mental health services
Weak mental health surveillance and fragmented health information systems	AI-enabled predictive analytics and surveillance	Apply machine learning to routine and community-level data to detect trends, geographic disparities, and unusual changes and to forecast service and workforce needs	Strengthened surveillance and more informed planning and resource allocation
Limited capacity for suicide risk assessment and relapse prediction	Machine learning–based risk prediction	Analyze multiple clinical and psychosocial variables to estimate risks of suicide, treatment non-adherence, relapse, or deterioration and prompt professional review. Predictions require local validation and reliable referral pathways	Improved risk identification and allocation of specialist resources
Scarcity of locally generated mental health evidence	AI-supported research and data analytics	Use machine learning and natural language processing to identify complex patterns and risk factors, analyze qualitative text, classify evidence, and develop predictive epidemiological models	Strengthened research capacity and evidence generation
Limited health-system efficiency	AI-assisted administrative and workflow support	Use natural language processing to generate clinician-reviewed documentation, predictive models to identify follow-up or non-attendance risks, and optimization algorithms to support scheduling and resource allocation	Reduced administrative burden and potentially improved workflow efficiency
Inequitable access to mental health services	AI-supported population health planning	Apply machine learning to demographic, geographic, and service-use data to identify underserved populations, forecast workforce and service needs, and model resource-allocation options	More equitable and evidence-informed mental health-service planning

The applications and potential impacts presented in this table are prospective and should not be interpreted as established outcomes in Somalia. Their feasibility, effectiveness, safety, acceptability, equity implications, and cost-effectiveness require evaluation through locally validated pilot studies and implementation research.

## AI-assisted mental health screening and early detection

Early recognition of mental disorders is essential for improving clinical outcomes. In Somalia, low mental health literacy, stigma, limited routine screening in primary care, and workforce shortages may delay identification (11–13). Digital platforms could supplement existing screening by administering validated instruments such as the Patient Health Questionnaire (PHQ-9), Generalized Anxiety Disorder-7 (GAD-7), Edinburgh Postnatal Depression Scale (EPDS), and PTSD screening tools (37, 38).

Mobile and web-based platforms could facilitate culturally adapted screening, automated scoring, symptom-severity stratification, triage, and referral based on predefined clinical protocols (19, 37, 38). These functions constitute conventional digital health; AI would be involved where machine-learning or natural language processing methods analyze multiple clinical inputs or free-text responses to support risk stratification and referral prioritization. Individuals requiring further evaluation could then be referred to primary care providers or mental health specialists for comprehensive clinical assessment (16, 19). Potential settings for supervised screening include primary health-care facilities, schools, universities, maternal health clinics, refugee settlements, and humanitarian settings, potentially facilitating earlier identification and more efficient referral workflows.

## Clinical decision support for non-specialist health care providers

Somalia's severe shortage of psychiatrists means that mental health care is often delivered by neurologists, general practitioners, primary care physicians, nurses, and community health workers (13, 31, 32). Task-sharing can expand services (39, 40), but non-specialist providers may have limited psychiatric training (11) and require additional clinical decision support and supervision, particularly for complex conditions (23).

AI-assisted clinical decision support systems could use machine learning or natural language processing to analyze structured clinical information and patient-reported or free-text symptoms, identify clinically relevant patterns, and generate individualized suggestions for differential diagnosis, risk assessment, management, and specialist or emergency referral (41–43). These functions differ from conventional rule-based systems that apply predefined clinical algorithms. Integration with recognized clinical guidance may help standardize care and reinforce evidence-based practice without displacing professional judgment (42, 44).

## Expanding access through telepsychiatry

Geographic disparities limit timely mental health care in rural, remote, and conflict-affected areas through logistical, infrastructural, and security barriers (12, 13, 31, 45). Telepsychiatry, which is a digital health service rather than an AI application, may reduce some geographic and logistical barriers (46). AI could enhance its delivery through clinical decision support, automated analysis of remote-monitoring data, and personalized care (47).

Conventional digital functions may include automated scheduling and longitudinal symptom monitoring, whereas AI-specific functions may include machine-learning-based referral prioritization, natural-language consultation summaries and translation, and detection of patterns indicating clinical deterioration (47). Such AI-assisted monitoring could support timely intervention and potentially reduce avoidable hospital admissions (16, 48). Together, these digital and AI-enabled functions may improve continuity of care and enable more efficient use of Somalia's limited psychiatric workforce.

## Conversational AI and digital mental health interventions

Advances in natural language processing have enabled conversational AI to interpret user input and generate context-sensitive responses for psychoeducation, symptom monitoring, behavioral activation, stress management, and basic cognitive behavioral therapy (CBT)-informed interventions (49). Although not substitutes for psychiatric care, these tools may offer low-intensity psychological support for mild to moderate symptoms, particularly where access to mental health professionals is limited (50, 51).

In Somalia, culturally adapted conversational AI could use natural language processing to understand Somali-language input and tailor responses to users' expressed needs. Potential functions include providing education, addressing mental health misconceptions, encouraging help-seeking, reinforcing medication adherence, promoting sleep hygiene, and supporting evidence-based self-management. Some of these functions could also be delivered through conventional digital platforms; the specific AI component is the interpretation of natural-language input and generation of adaptive, context-sensitive responses.

Privacy-preserving platforms may reduce stigma-related barriers by providing private access to mental health information and initial psychological support before engagement with formal services (52). These tools should incorporate clear crisis-response protocols and pathways for timely referral to qualified professionals when higher-level care is required (53).

## Predictive analytics for suicide prevention and early intervention

Suicide risk prediction represents an important yet ethically sensitive application of AI in psychiatry. Machine-learning models may identify individuals at elevated suicide risk by integrating clinical, behavioral, psychosocial, and digital data (16, 54, 55). Although these models could facilitate earlier identification of individuals requiring intensive assessment or follow-up, their predictive accuracy and calibration may vary across populations and clinical settings (54). False-positive results may cause unnecessary distress or inappropriate intervention, whereas false-negative results may provide false reassurance and delay needed care. Accordingly, clinical use in Somalia would first require evidence of acceptable predictive performance and calibration in local populations.

In Somalia, locally validated machine-learning models may help clinicians identify high-risk individuals, prioritize follow-up, and inform the allocation of scarce mental health resources. Similar models may support the assessment of relapse, treatment response, and clinical deterioration among individuals with severe mental illness, potentially enabling earlier intervention and improved continuity of care (16). Their clinical usefulness would depend on reliable referral and follow-up pathways. These systems may inform clinical assessment, but responsibility for clinical decision-making must remain with qualified professionals.

In low-connectivity or insecurity-affected settings, conversational and predictive AI tools should incorporate low-bandwidth or offline safety information and locally verified, adaptable referral pathways. Reliable crisis-response, referral, and follow-up pathways are prerequisites for their clinical usefulness. High-risk alerts should prompt review by a trained professional rather than autonomous action, with alternative community-based contacts or safely accessible facilities identified when real-time communication or the primary referral site is unavailable. Users should also be clearly informed when immediate escalation cannot be guaranteed.

## AI-enabled population-level mental health surveillance and health-system planning

Reliable population-level mental health information is essential for effective policy and planning, yet routine surveillance remains limited in Somalia (11, 31). As digital information systems develop, aggregated or appropriately de-identified data from electronic health records, community health programmes, humanitarian health information systems, and national surveys could support analysis of disease patterns, service utilization, and unmet needs. AI-specific analytical methods could then assist with pattern detection and forecasting across these population-level datasets (18, 56).

Where data quality and representativeness are adequate, advanced analytics may help identify geographic disparities in service availability, detect changes in psychological distress following humanitarian emergencies, forecast workforce needs, and inform resource allocation (56). This population-level surveillance function is distinct from the monitoring, risk classification, or profiling of identifiable individuals, which raises more direct concerns regarding consent, confidentiality, clinical accountability, and potential misuse. These activities should have clearly defined purposes, separate access controls, and appropriate accountability mechanisms.

## Accelerating mental health research and innovation

AI could support mental health research in Somalia through machine-learning analysis of large epidemiological datasets to identify complex patterns and previously unrecognized risk factors, develop predictive models, and inform precision public health approaches. Natural language processing could assist with the classification and analysis of qualitative text and the identification and synthesis of emerging evidence relevant to local priorities (57, 58).

These applications depend on high-quality, representative datasets reflecting Somalia's linguistic, cultural, and epidemiological diversity. Investment in digital infrastructure and standardized data systems, together with interdisciplinary collaboration among clinicians, data scientists, public health researchers, and policymakers, would be needed to support scientifically rigorous, ethically responsible, and locally relevant applications (59, 60).

Collectively, these applications illustrate how AI could strengthen prevention, early detection, clinical care, workforce support, research, surveillance, and policy development in Somalia. AI should complement conventional health system strengthening by supporting efforts to expand the mental health workforce, integrate mental health into primary care, strengthen digital infrastructure, and develop community-based services (20, 33). Clinical responsibility for diagnosis, treatment, referral, and patient safety must remain with qualified professionals. Translating this potential into practice will depend on responsible deployment, clear accountability, strengthened local capabilities, and sustained evaluation (20).

Given Somalia's current health-system capacity, three applications may warrant priority for near-term pilot testing: AI-assisted screening and referral support in primary and community care, mhGAP-aligned clinical decision support for non-specialist providers, and AI-enhanced telepsychiatry.

## Challenges, ethical considerations, and implementation barriers

Despite its potential, AI cannot overcome the structural challenges facing Somalia's mental health system without a supportive digital ecosystem, high-quality data, a trained workforce, and sustained financial investment (20). Without these prerequisites, AI may exacerbate health inequities, compromise privacy, and undermine trust in health systems rather than achieve its intended benefits (34). Addressing these implementation and ethical challenges is therefore essential for safe, equitable, and responsible AI integration (20).

Specific unintended consequences include algorithmic bias, overreliance on AI, and digital exclusion. Models developed from non-representative data may perform unevenly across linguistic, cultural, demographic, or geographic groups, resulting in biased assessment or referral (48), while overreliance on algorithmic outputs may weaken clinical judgment (61). Digital delivery may also exclude people with limited connectivity, device access, or digital literacy, particularly rural communities, older adults, socioeconomically disadvantaged groups, and other marginalized populations (62). Mitigation measures should include locally representative validation, performance assessment across population subgroups, clinician training, and continued monitoring for inequitable outcomes.

## Digital infrastructure and technological readiness

AI-enabled mental health interventions depend on reliable digital infrastructure. Internet connectivity and smartphone use have expanded in Somalia (63), but substantial urban–rural disparities persist in internet access, electricity, digital literacy, and availability of digital health technologies (45, 64). Somalia's current health information system remains at an early and uneven stage of digital maturity, with substantial variation across facilities and regions and continued reliance on paper-based records in many settings (65). Readiness for AI-enabled applications should be assessed using indicators such as electricity availability and continuity, internet coverage and reliability, access to suitable devices, digital literacy, electronic-record adoption, interoperability, data completeness and timeliness, cybersecurity capacity, and availability of technical support. Facility-level readiness assessments are needed because national averages may conceal substantial differences between urban, rural, and conflict-affected settings.

## Data availability, quality, and algorithmic bias

AI systems require large, high-quality, and representative datasets for development, validation, and generalizability. Somalia lacks comprehensive mental health databases, nationwide interoperable digital records, and nationally representative datasets, limiting the standardized data needed to develop and validate robust AI models (31, 65). A further concern is that many existing AI models are developed using high-income-country data, raising concerns about their generalizability to Somalia's distinct cultural, linguistic, and epidemiological context (58).

For example, language-based models may perform inconsistently in digitally underrepresented languages such as Somali and may not adequately capture culturally specific Somali expressions and idioms of psychological distress (66). Similarly, AI models that perform well on internal datasets may not retain comparable performance when applied to different patient populations or clinical settings, underscoring the importance of external validation using locally representative data (67). Models trained on non-representative data may produce biased predictions, reduce diagnostic performance, and exacerbate existing mental health inequities (24). Locally representative datasets and context-specific validation will therefore be essential before widespread clinical implementation. Accordingly, the proposed AI applications should be regarded as prospective, and their feasibility, effectiveness, safety, acceptability, equity, and cost-effectiveness should be evaluated in the Somali context before broader implementation.

## Privacy, confidentiality, and cybersecurity

Mental health information is highly sensitive, and AI applications often require extensive collection, storage, processing, sharing, and transmission of patient data, raising concerns about confidentiality, informed consent, data ownership, privacy, and cybersecurity (24, 53). Where legal protections for digital health data are underdeveloped, unauthorized disclosure or misuse may expose individuals to stigma, discrimination, and other adverse social consequences (68).

In fragile governance contexts, mental health data and AI-enabled technologies could be repurposed for non-clinical surveillance, profiling, or population tracking without adequate safeguards or meaningful informed consent, posing particular risks to vulnerable populations with limited control over how their personal health information is collected, shared, and used. AI-enabled mental health initiatives should therefore incorporate clear purpose limitations, data minimization, restricted access, independent oversight, secure storage, transparent data-sharing policies, and robust cybersecurity measures to protect privacy and maintain public trust (18, 24).

## Ethical considerations and clinical accountability

Psychiatric decision-making requires nuanced assessment of emotional, behavioral, cultural, and social factors that algorithms may not fully capture. Uncritical reliance on AI recommendations could lead to diagnostic errors, inappropriate treatment, and diminished clinical autonomy (24, 69). Limited interpretability presents an additional concern because many advanced AI systems operate as “black boxes,” limiting transparency in how recommendations are generated (24, 70). Ensuring explainability, transparency, fairness, and human oversight are therefore essential (24). AI should function as decision support while preserving clinical expertise, professional judgment, and therapeutic relationships of qualified mental health professionals (69). These recommendations align with WHO guidance and the AU Continental AI Strategy, which emphasize human autonomy and oversight, human rights and dignity, transparency, accountability, inclusion, and equity (18, 71).

## Regulatory and governance challenges

Effective AI implementation requires governance frameworks establishing standards for safety, quality, accountability, and ethical practice (24). Somalia currently has limited health-care-specific AI regulation (20). Policies should address algorithm validation, clinical approval, liability, procurement, data protection, and post-implementation monitoring to ensure safe, effective, and equitable use. Governance should adopt a risk-based, lifecycle approach aligned with WHO guidance and the AU Continental AI Strategy, including independent evaluation, stakeholder participation, and mechanisms for monitoring, accountability, and redress (18, 71). National governance should also foster collaboration among policymakers, clinicians, researchers, legal experts, technologists, and patient representatives to guide responsible innovation and protect patient rights (72).

## Workforce capacity and AI literacy

Successful AI adoption requires both technological readiness and a prepared health workforce. Many clinicians and health-care administrators have limited AI literacy and formal training in AI concepts, digital health technologies, and the interpretation and integration of AI-generated recommendations into clinical practice (73, 74). Inadequate training may lead to either underuse of beneficial tools or overreliance on algorithmic outputs. Building AI literacy through undergraduate and postgraduate education, continuing professional development, and interdisciplinary collaboration will be essential for responsible adoption while preserving clinical judgment and patient-centered care (58, 68).

## Sociocultural acceptability and equity

AI-enabled mental health interventions must be acceptable to the communities they serve. Somalia's cultural diversity, linguistic characteristics, religious values, and help-seeking patterns should inform the design and implementation of AI models (58). Because Somali remains digitally underrepresented (66), limited annotated mental health data, dialectal variation, and culturally specific terminology may reduce the accuracy of language-based models. Cultural and spiritual explanations of distress, including beliefs involving jinn or sihr, may also influence how symptoms are described, understood, and presented for care (12, 13). AI systems should recognize these explanatory frameworks respectfully without treating them as clinical diagnoses or allowing them to delay appropriate assessment and referral. Failure to account for local culture and language may limit uptake and compromise assessment accuracy (24, 58). Unequal access to smartphones, internet connectivity, and digital literacy may also exclude rural populations, older adults, and socioeconomically disadvantaged communities, potentially reinforcing existing inequities. Equity considerations should specifically address internally displaced persons, adolescents, women, rural communities, and people with severe mental illness. Their differing needs relating to digital access, privacy, safeguarding, referral pathways, continuity of care, and clinical oversight should inform co-design, pilot testing, and evaluation to avoid reinforcing existing disparities. Culturally sensitive co-design should involve patients, families, clinicians, community leaders, and, where appropriate, trusted traditional and religious healers to inform acceptable language, community safeguards, and referral pathways while maintaining professional clinical oversight (58).

## Financial sustainability, political economy, and security

Although AI may improve efficiency, implementation requires substantial investment in digital infrastructure, workforce training, software development, cybersecurity, maintenance, and evaluation (20). Financing should progressively incorporate dedicated domestic budget lines within national mental health and digital health plans, complemented by coordinated donor and development-partner support (68). A Ministry of Health-led coordination mechanism should align funders around common priorities, interoperability and procurement standards, reporting requirements, and sustainability plans to reduce duplication, fragmented pilots, and vendor dependence. Economic evaluation should be embedded prospectively within pilot programmes and assess start-up and recurrent costs, total cost of ownership, cost-effectiveness, budget impact, affordability, opportunity costs, and the equitable distribution of benefits before broader implementation.

Implementation may also be affected by competing political priorities, fragmented institutional responsibilities, limited regulatory and procurement capacity, and dependence on external funding (68). Conflict and insecurity may disrupt electricity and connectivity, restrict access to facilities, interrupt supervision and referral pathways, and threaten the continuity of services. Implementation plans should therefore incorporate context-specific political and security risk assessments, flexible geographic sequencing, contingency arrangements, and coordination with local authorities, communities, and humanitarian partners. Long-term sustainability will depend on national ownership, coordinated financing, institutional capacity, and adaptation to changing security and service-delivery conditions.

## Policy roadmap and future directions

Realizing AI's potential in Somalia's mental health system will require a coordinated, phased, and context-specific approach aligned with broader health system strengthening (68). AI should be integrated into national mental health policies, digital health strategies, and universal health coverage initiatives rather than implemented as a standalone solution. Sustainable implementation will require political commitment, multisectoral collaboration, ethical governance, and continuous evaluation to improve access, quality, equity, and efficiency of mental health care.

## A phased roadmap for AI implementation in Somalia

AI integration into Somalia's mental health system should follow a gradual, evidence-informed approach rather than immediate nationwide deployment. Given limited digital infrastructure, workforce capacity, regulatory readiness, and locally generated evidence, phased implementation would allow stakeholders to establish essential foundations, evaluate AI under real-world conditions, strengthen institutional capacity, and refine strategies before national scale-up (68). Advanced, data-intensive AI applications should be considered only after reliable digital records, interoperable information systems, representative local data, and appropriate governance mechanisms have been established. Because reliable national baseline data remain limited, phase-specific numerical targets should be established through baseline assessments and defined prospectively before implementation. Figure 1 presents the proposed five-phase roadmap and indicative milestones for responsible AI integration into Somalia's mental health system.

**Figure 1:**
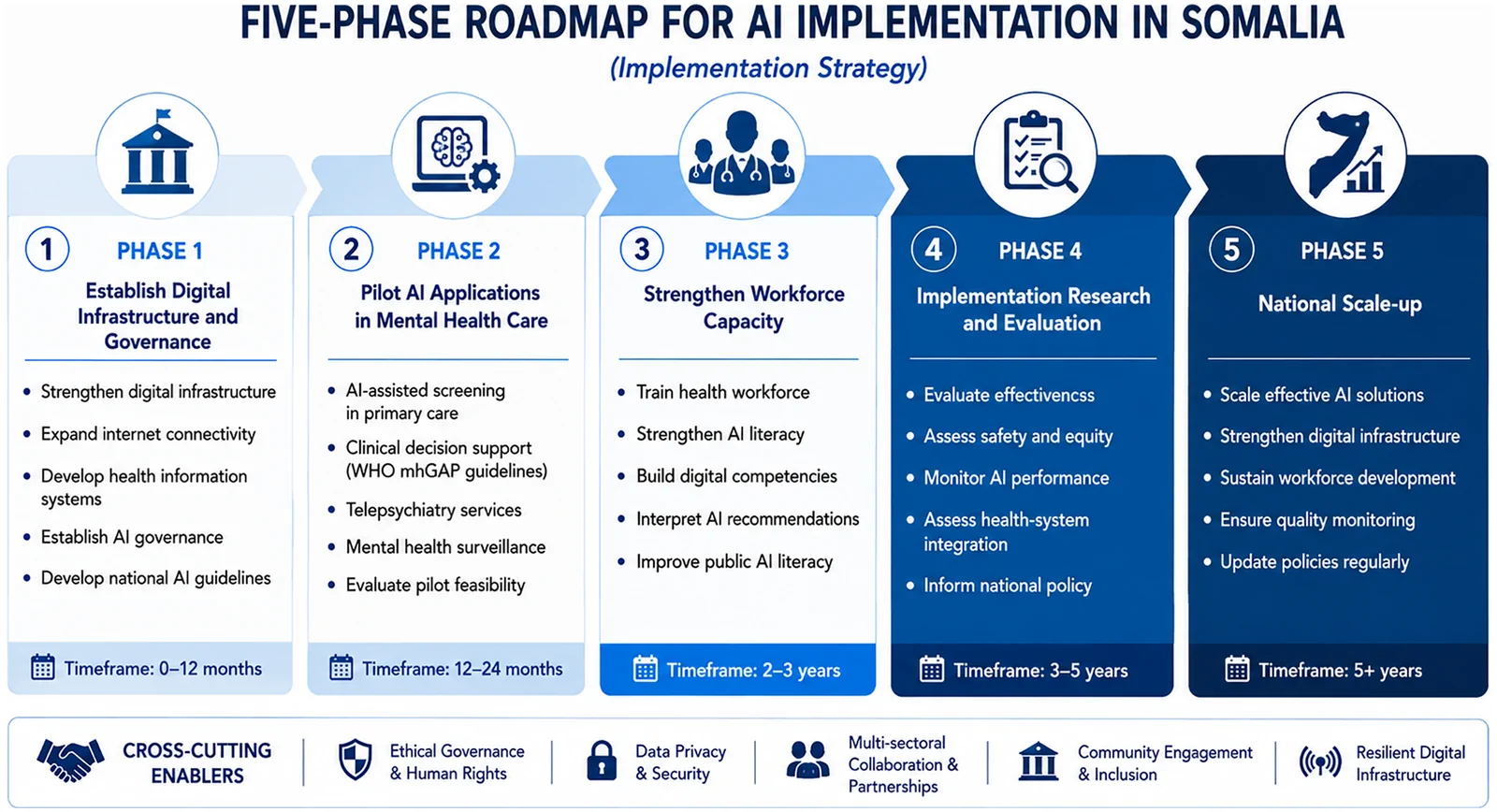
Proposed five-phase roadmap for implementing artificial intelligence in Somalia's mental health system. The timeframes are indicative rather than fixed and should be adapted according to baseline readiness, resource availability, security conditions, and evidence generated during preceding phases. Phases may overlap, and progression to broader implementation should depend on achieving predefined milestones and demonstrating feasibility, safety, effectiveness, acceptability, equity, and sustainability. Ethical governance and human rights, data privacy and security, multisectoral collaboration and partnerships, community engagement and inclusion, and resilient digital infrastructure are cross-cutting enablers applicable throughout all phases.

## Phase 1: establish digital infrastructure and governance

The first phase should establish the foundations for AI implementation by strengthening digital health infrastructure, internet connectivity, electronic health record (EHR) systems, and interoperability across health-care facilities. In parallel, national governance and regulatory frameworks should address data governance, privacy, cybersecurity, informed consent, algorithm transparency, accountability, clinical oversight, procurement standards, and post-implementation monitoring to support the safe, ethical, and responsible use of AI (58, 74). These frameworks should also safeguard patient autonomy and ensure meaningful participation of patients, clinicians, and communities in the design, evaluation, and governance of AI-enabled mental health services. The Ministry of Health should lead national priority-setting, regulation, stakeholder coordination, and oversight. Professional associations should define clinical and competency standards; academic institutions should lead local research, validation, training, and independent evaluation; and international partners should provide coordinated technical and financial support aligned with national priorities while avoiding parallel systems and long-term dependency. Indicative milestones include: (1) the proportion of participating facilities meeting predefined electricity and internet reliability standards; (2) the proportion using interoperable EHRs compliant with national data standards; and (3) formal adoption of national AI governance guidelines and designation of an accountable oversight body.

## Phase 2: pilot AI applications in mental health care

Once foundational infrastructure and governance are established, AI should be introduced through carefully designed pilots in selected health-care facilities. Priority applications should include AI-assisted screening in primary care, clinical decision support based on WHO mhGAP guidelines, telepsychiatry for underserved communities, and AI-supported mental health surveillance. These pilots should assess technical feasibility, clinical usefulness, operational challenges, and user acceptance within the Somali context to inform decisions regarding their suitability for possible broader deployment (68). Indicative milestones include: (1) completion of pilots across selected urban and rural facilities; (2) achievement of predefined feasibility and operational targets, such as system reliability, successful task completion, and integration into clinical workflows; and (3) achievement of application-specific thresholds for clinical performance, safety, user acceptability, and referral completion where applicable.

## Phase 3: strengthen workforce capacity

AI implementation requires a workforce capable of using digital health technologies safely and effectively. Training should target psychiatrists, primary care physicians, nurses, psychologists, community health workers, health informatics professionals, and health-care administrators, with the aim of strengthening AI literacy, digital competencies, and interpretation of AI-generated recommendations while emphasizing AI as decision support rather than a substitute for clinical judgment (73, 74). Public education should also aim to strengthen digital and mental health literacy and support the informed and appropriate use of AI-enabled mental health services. Indicative milestones include: (1) the number and proportion of targeted professionals completing training; (2) the proportion achieving a predefined competency standard; and (3) the proportion receiving continuing supervision or refresher training.

## Phase 4: conduct implementation research and evaluation

Before nationwide implementation, AI interventions should be rigorously evaluated for effectiveness, safety, acceptability, equity, and cost-effectiveness in the Somali context. Implementation research should assess clinical outcomes, patient and provider experiences, algorithm performance, health-system integration, and unintended consequences. Evaluation findings should be disaggregated by population subgroup and geographic setting to identify differences in access, performance, acceptability, safety, and potential harms. Continuous monitoring and iterative refinement should aim to support accuracy, cultural appropriateness, and responsiveness to evolving health-care needs, while the resulting evidence could inform national policy and future implementation decisions. Indicative milestones include: (1) achievement of prespecified effectiveness, algorithm-performance, and safety thresholds; (2) predefined levels of patient and provider acceptability; and (3) assessment of costs and differences in access, performance, and harms by geographic setting, sex or gender, age, and displacement status.

## Phase 5: scale successful interventions nationally

AI applications demonstrating effectiveness, safety, acceptability, equity, and long-term sustainability could be considered for progressive integration into Somalia's national mental health system. Any coordinated scale-up should maintain investment in digital infrastructure, workforce development, cybersecurity, and health information systems (68). Ongoing monitoring, quality assurance, and periodic policy review would be necessary to evaluate and support potential improvements in access, quality, efficiency, and equity while adapting to technological advances and evolving population health needs. Indicative milestones include: (1) the proportion of eligible facilities and regions adopting validated interventions; (2) sustained system reliability, workforce competency, and referral completion after scale-up; and (3) periodic safety and equity audits demonstrating that implementation has not widened disparities across population groups or geographic settings.

## Monitoring and evaluation

Monitoring and evaluation should be integrated throughout implementation, with baseline measures established before pilot testing. Key indicators should include service reach and uptake; screening, referral, and follow-up completion; clinical outcomes and patient safety; algorithmic accuracy, calibration, and false-positive and false-negative rates; usability and acceptability among patients and providers; effects on workload and service efficiency; system reliability and data security; and start-up and recurrent costs. Indicators should be disaggregated by sex, age, geographic location, displacement status, and other relevant characteristics to assess equity. Progression between phases should depend on meeting predefined, context-appropriate performance and safety criteria.

## Limitations

As a Perspective, this article provides a selective synthesis of relevant literature rather than an exhaustive systematic review or formal appraisal of evidence certainty. The Somalia-specific evidence base is limited in scope and representativeness, and evidence supporting several proposed AI applications is derived from substantially different health-system contexts. The anticipated impacts and phased roadmap are therefore context-informed strategic proposals rather than empirically validated outcomes or implementation models. Moreover, the roadmap was developed without formal stakeholder consultation or a structured consensus-building process and should be regarded as a preliminary framework rather than an agreed national implementation plan. The proposed applications and potential benefits should not be assumed to be transferable to Somalia without local research, validation, and pilot testing, followed by continued adaptation to evolving health-system, resource, and security conditions.

## Future directions

Future efforts should generate robust evidence for safe and effective AI integration into Somalia's mental health system. Priorities include developing and validating models using representative Somali datasets; evaluating clinical effectiveness, cost-effectiveness, equity, and implementation feasibility; and strengthening digital infrastructure and interoperable health information systems. Interdisciplinary collaboration among clinicians, researchers, data scientists, engineers, policymakers, community representatives, and regional and international partners will be essential to advance locally led research and contextually relevant innovation. Capacity building should combine interdisciplinary training and mentorship with sustained investment in Somali universities, hospitals, research, and data infrastructure. People with lived experience, caregivers, and community and religious leaders should be meaningfully engaged in priority-setting, study co-design, interpretation of findings, and implementation. Continuous monitoring should be prioritized to ensure that AI remains safe, transparent, equitable, and responsive to evolving population health needs.

## Conclusion

AI is not a substitute for the sustained investment, workforce development, primary care integration, and community-based services required to strengthen mental health care in Somalia. Nevertheless, when carefully adapted and governed, it may support earlier identification, non-specialist providers, remote care, routine data use, and health-system planning.

Its contribution will depend on phased, locally validated implementation supported by appropriate safeguards and continued evaluation. By treating AI as a supportive component of broader health-system reform rather than a standalone solution, Somalia can explore its potential while minimizing harm and generating relevant lessons for other fragile and resource-constrained settings.

## Data Availability

The original contributions presented in the study are included in the article/Supplementary Material, further inquiries can be directed to the corresponding author.
